# The pharmacological and functional characterization of the serotonergic system in *Anopheles gambiae* and *Aedes aegypti*: influences on flight and blood-feeding behavior

**DOI:** 10.1038/s41598-019-38806-1

**Published:** 2019-03-14

**Authors:** Michelle Ngai, Douglas A. Shoue, Zoe Loh, Mary Ann McDowell

**Affiliations:** Eck Institute for Global Health, Department of Biological Sciences, University of Notre Dame, Notre Dame, IN 46656 USA

## Abstract

*Aedes aegypti* and *Anopheles gambiae* harbor the causative agents of diseases such as dengue fever and malaria, afflicting human morbidity and mortality worldwide. Given the worldwide emergence of resistance to insecticides, the current mainstay for vector control, identification of alternative modes of action for future insecticides is paramount. The serotonergic (5-HT) system has been documented to impact physiological mechanisms involved in disease transmission, suggesting its potential as a new mode of action target for future insecticide development. Target 5-HT receptors were cloned and expressed in the HEK293 cell line for functional and pharmacological characterization. Manipulation of the 5-HT system through microinjection of compounds suggests its involvement in the modulation of flight performance and blood-feeding behavior. By attenuating these two determinants of vectorial capacity, transmission and burden of disease could effectively be reduced. Considering these positive global health implications, the 5-HT system is a compelling target for the novel insecticide pipeline.

## Introduction

Mosquitoes harbor multiple causative agents of diseases pertinent to global health. *Aedes aegypti* and *Anopheles gambiae*, specifically, transmit the parasite and arboviruses responsible for malaria, dengue, Zika, and chikungunya, afflicting high human morbidity and mortality worldwide. Globalization and climate change have been major drivers behind the geographic expansion of certain mosquito species, resulting in the global spread of mosquito-borne diseases^1^. Considerable efforts have been placed on an integrative disease control strategy, though the use of chemical compounds has been heavily relied upon for vector control. Insecticide resistance, however, has developed to commonly used classes of compounds, most notably pyrethroids, the only class appropriate for close contact with humans^2,3^. In light of the reduced efficacy of current insecticides, there has been a renewed vigor in the identification of novel modes of action for insecticidal compounds in the development pipeline. G-protein coupled receptors (GPCRs), known to mediate a broad spectrum of physiological and behavioral processes in insects, are considered to be highly “druggable” targets and focus has thus shifted towards this large cell-surface protein family^4–8^. Given the current technology for next generation sequencing, high-quality assemblies of arthropod genomes including *Ae aegypti* and *An. gambiae* have increased, facilitating the identification of GPCRs for functional and pharmacological investigation.

Biogenic amine receptors are classified as Class A GPCRs, and traction is gaining in examining their potential as pesticide targets. Mosquito vectors have orthologs of mammalian biogenic amine receptors including serotonin, dopamine, octopamine/tyramine, and muscarinic acetylcholine receptors. Localization of the serotonin (5-HT) receptor family suggests its role in mediating processes important for disease transmission, including blood-feeding and locomotion, making this family of particular interest. The distribution network of serotoninergic nerves is extensive; though primarily within the central nervous system, studies describe branching into the peripheral nerves and organs^9–11^. High expression levels of 5-HT receptors and the presence of 5-HT immunoreactive nerves within the salivary gland are indicative of its role in salivation, an integral component to host-seeking behavior, and consequently, blood feeding, duration of probing, and oviposition^12–14^. A previous study also implicated the serotonergic system in the regulation of locomotion activity due to the expression patterns of 5-HT_2_ receptors in the midline motor neurons of *D. melanogaster*^15^. Female *Ae. aegypti* and *An. gambiae* mosquitoes are predicted to take a blood meal every two to three days to ensure availability of proteins crucial for the egg development process^16^. Motor activity is relevant to blood meal acquisition, enabling travel to food sources and oviposition sites. Disruption to either of these physiological responses has the potential to negatively impact vectorial capacity and effectively reduce disease transmission and burden. Therefore, as indirect determinants of vectorial competence, disrupting feeding and flight via the 5-HT receptor family could prove to be a new avenue and mode of action target for insecticide development.

## Results

### Bioinformatics

**Figure 1:**
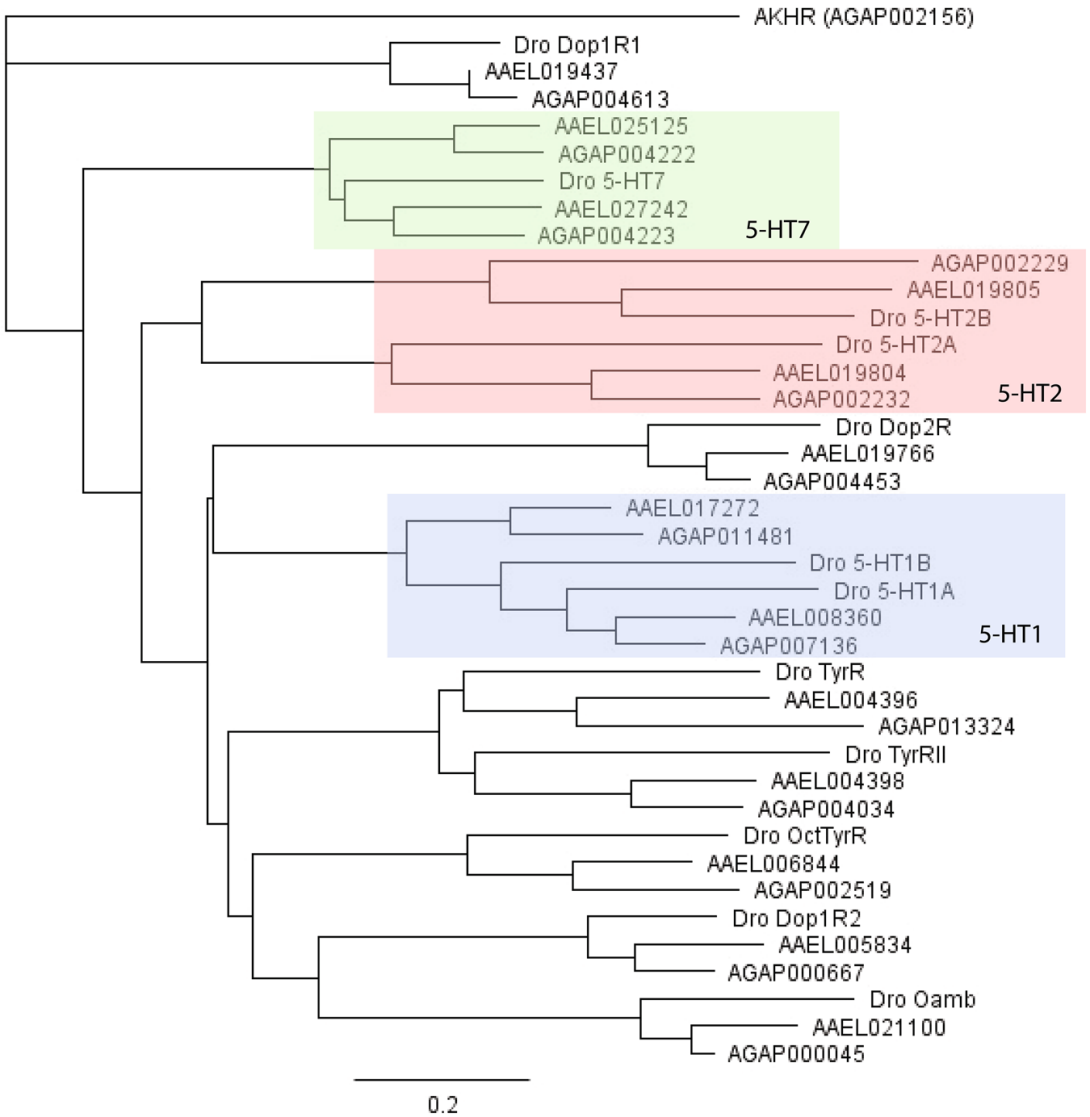
Phylogenetic tree of biogenic amine receptors in *Ae. aegypti, An. gambiae and D. melanogaster*. The rooted phylogenetic tree was built with putative 5-HT receptor and other biogenic amine receptor amino acid sequences in *Ae. aegypti* (AAEL), *An. gambiae* (AGAP), and *D. melanogaster* (Dro) species; the *An. gambiae* Adipokinetic Hormone receptor was the outgroup. Receptors are denoted only by their unique accession number. The blue, red, and green clusters represent the various 5-HT subfamilies; the blue includes proteins most similar to the 5-HT_1_ subfamily, while the red and green encompass those grouped into the 5-HT_2_ and 5-HT_7_ subfamilies, respectively.

Putative 5-HT GPCR genes were mined in VectorBase; eight *Ae. aegypti* and six *An. gambiae* genes were discovered to have distinct transcripts. A rooted phylogenetic tree was constructed using Geneious version 5.1.5 (http://www.geneious.com)^17^ with the amino acid sequences of the putative 5-HT receptors and other biogenic amine receptor families in *Ae. aegypti* (AAEL), *An. gambiae* (AGAP), and *D. melanogaster* (Dro) species. Utilizing the *An. gambiae* Adipokinetic Hormone receptor protein (AGAP002156) as the outgroup, the 5-HT receptors were grouped into distinct clades, highlighting their respective subfamilies (Fig. 1). *Ae. aegypti* proteins (AAEL008360 and AAEL017272) and *An. gambiae* proteins (AGAP007136 and AGAP011481) clustered with the *D. melanogaster* 5-HT_1_ receptor family, illustrating sequence homology and their potential functional similarity via the Gα_i/o_ protein and consequently, inhibition of cyclic AMP. Similarly, AAEL019804, AAEL019805, AGAP002232, and AGAP002229 were grouped with the *D. melanogaster* 5-HT_2_ receptor family, which signals through the Gα_q/11_ protein, ultimately increasing intracellular calcium levels. Finally, AAEL025125, AAEL027242, AGAP004222, and AGAP004223 proteins exhibited homology to the *D. melanogaster* protein within the 5-HT_7_ subfamily, suggesting a coupling to the Gα_s_ protein and increased production of cAMP levels upon activation.

### Expression analysis

**Figure 2:**
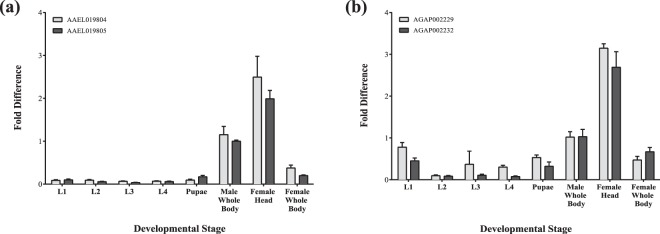
Expression profile of the *Ae. aegypti* and *An. gambiae* 5-HT_2_ receptor family. Quantitative RT-PCR was conducted on the immature and mature life stages with SYBRGreen for the 5-HT2 genes. The expression level of the genes was determined via comparative quantification. Housekeeping gene, 40S ribosomal protein S7 (AAEL009496 and AGAP010592) was utilized as internal controls. Fold change ± SEM compared to the male whole body is presented. Two independent experiments were each performed with three technical replicates.

Insecticides disrupting the central nervous system, such as pyrethroids, have proven to be effective, prompting our focus on identifying other targets that have a similar ability to alter neurocognitive function. Overall, there is considerable expression of the 5-HT_2_ receptor genes in the female head, demonstrating the possibility of targeting nervous tissue for the development of a novel insecticide (Fig. 2). Additionally, the expression profile generally shows very low or absent levels of 5-HT_2_ transcripts in the immature stages, suggesting that concentrating on the adult mosquito could potentially be more fruitful.

### Functional characterization

**Figure 3:**
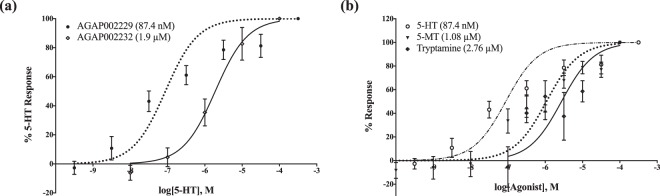
Dose-dependent calcium response in CRE-*luc2P* HEK-293 cells independently expressing AGAP002229 or AGAP002232 via Fluo-4 calcium assays. (**a**) The EC50 for AGAP002229 is 87.4 nM while the EC50 for AGAP002232 is 1.9 μM. Legend includes gene name (EC50). (**b**) HEK-293 cells expressing AGAP002229 were presented with agonists. EC50 for 5-MT is 1.08 μM while tryptamine’s EC50 is 2.76 μM. Legend includes agonist (EC50). EC50 ± standard error mean is represented. Three independent experiments were each completed with three technical replicates.

The cDNA for AGAP002232 and AGAP002229 encoded proteins 825 and 911 amino acids in length, respectively. Through the Fluo-4 calcium assay, a flux in intracellular calcium levels was observed in both cell lines in response to 5-HT, indicating signaling through the Gα_q/11_ protein^18^. Activity levels altered in a dose-dependent manner for both cell lines with 5-HT concentrations ranging between 10^−10^ to 10^−4^ M; increasing concentrations of 5-HT resulted in higher intracellular calcium levels until a saturation point was reached (Fig. 3a). The receptor product of AGAP002229 is more sensitive to 5-HT than AGAP002232 with half maximal effective concentrations (EC_50_) of 87.4 nM and 1.9 µM, respectively (p < 0.0001). The pharmacological profile for both AGAP002229 and AGAP002232 were also evaluated. Response to biogenic amines including octopamine, histamine, dopamine, and tyramine at 10^−4^ M was evaluated and indiscernible from background levels, indicating the receptors’ specificity to the 5-HT ligand (Supplementary Fig. S1). Agonist compounds, 5-methoxytryptamine and tryptamine, were able to activate AGAP002229 but not AGAP002232. Additionally, neither agonist induced calcium flux as potently as the native 5-HT ligand (p < 0.0001); the EC_50_ values were a few magnitudes of order higher (Fig. 3b).

**Figure 4:**
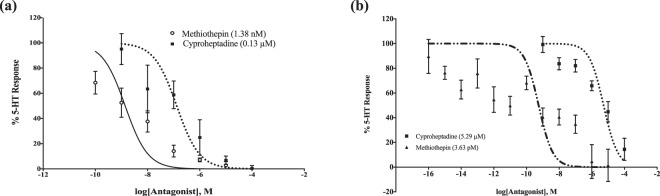
Dose-dependent calcium response to 5-HT and antagonists, cyproheptadine and methiothepin in CRE-*luc2P* HEK-293 cells expressing (**a**) AGAP002229 and (**b**) AGAP002232. AGAP002229: cyproheptadine IC50 is 0.13 μM, while methiothepin IC50 is 1.38 nM. AGAP002232: cyproheptadine IC50 is 5.29 μM, while methiothepin IC50 is 0.56 nM. IC50 ± standard error mean is represented. Three independent experiments were each conducted with three technical replicates. Legend includes compound (IC50 value).

Antagonist chemical compounds cyproheptadine, methiothepin, mianserin, spiperone, and yohimbine were all analyzed, but only the former two exhibited the ability to inhibit activity in a dose-response manner (Supplementary Fig. S2). The half maximal inhibition (IC_50_) of the calcium concentration reveals cyproheptadine is more effective in independently suppressing AGAP002229 (0.13 μM) versus AGAP002232 HEK-293 cells (5.29 μM) (p < 0.0001). Methiothepin proved to be a more potent antagonist than cyproheptadine, exhibiting inhibitory effects at concentrations as low as 10^−16^ M. Methiothepin inhibited AGAP002232 with an IC_50_ value of 0.56 nM (Fig. 4). Due to its robust effect on the 5-HT_2_ receptor and previous literature demonstrating methiothepin’s ability to inhibit D1-like dopamine receptors^19^, methiothepin was also assessed in HEK-293 cells expressing an octopamine receptor (AGAP000045) to determine its potential impact on other biogenic amine receptors^20^. A range of concentrations between 10^−10^ to 10^−4^ M was applied to the cell line to test methiothepin’s inhibitory effect; while there was marked inhibition at 10^−4^ M, all other concentrations did not elicit a similar result (Supplementary Fig. S3). To determine the specificity of these genes to the Gα_q/11_signaling pathway, GloResponse™ CRE-*luc2P* reporter cells that measure Gα_s_ stimulated cyclic AMP levels were utilized to measure the receptor response to 10^−4^ M serotonin under Gα_s_ and Gα_i/o_ conditions and resulted in no measurable responses (Supplementary Fig. S4).

### The serotonergic system and locomotion

**Figure 5:**
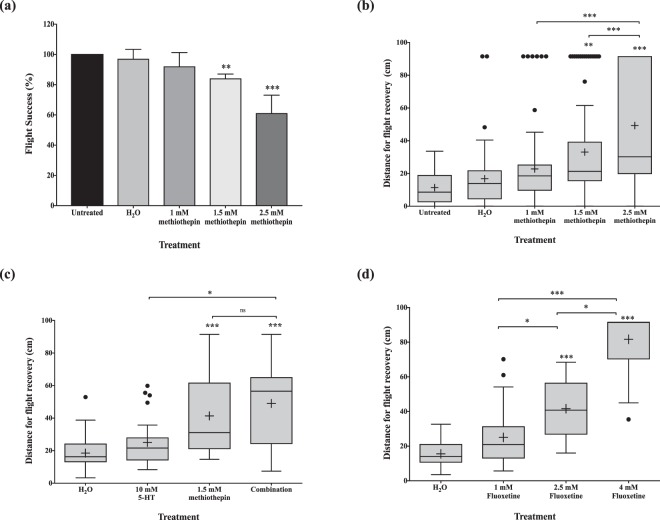
Locomotion and the serotonergic system in *Ae. aegypti*. (**a**) Flight success after microinjection. (**b**) Flight ability after treatment with methiothepin, a 5-HT receptor antagonist. (**c**) Flight success after treatment with methiothepin and 5-HT. (**d**) Flight ability post microinjection with fluoxetine, a selective serotonin-reuptake inhibitor. Three independent trials were conducted. Box plots represent the median value (line), mean value (+), interquartile range (box), and Tukey whiskers equivalent to 1.5 fold of the interquartile range. A distance of 91.5 cm was recorded for each mosquito that did not fly. ^•^Represents an individual mosquito outside the range of the Tukey whiskers. Data were compared with the Kruskal-Wallis and Dunnett’s multiple comparison tests after failing the Kolmogorov-Smirnov normality test. **p-value < 0.002; ***p-value < 0.0002.

Five- to seven-day old *Ae. aegypti* were microinjected and subjected to a flight cylinder in order to identify the serotonergic capacity on flight performance, a common proxy for locomotive ability^21^. In terms of the percentage of mosquitoes that were successfully able to fly, there was no significant difference between non-injected mosquitoes and the controls injected with ultrapure H_2_O (Fig. 5a). This result was further confirmed when the average distance of landed flight was calculated, suggesting that injections do not significantly impair flight performance; the average for the untreated versus H_2_O-treated mosquitoes were 11.39 cm and 16.78 cm, respectively. Based on the flight cylinder results, locomotion appeared to be impaired in a dose-dependent manner when independently injected with methiothepin. There was a reduction in successful flights with increasing doses of injected methiothepin. After treatment with 1 mM, 1.5 mM, and 2.5 mM methiothepin, we observed a decline in success with 92, 84, and 61%, respectively (Fig. 5a). Additionally, there was a distinct difference in the distance the mosquitoes dropped before flying and landing on the flight cylinder. While the H_2_O-treated average was 16.78 cm, the 1 mM, 1.5 mM, and 2.5 mM methiothepin-treated averages were 22.77 cm, 33.04 cm, and 49.27 cm, respectively, indicating locomotion was impaired with incremental doses of the drug (p < 0.0001) (Fig. 5b). Due to methiothepin’s strong antagonistic activity on the 5-HT_2_ receptor, we speculated that flooding the mosquitoes’ system with exogenous 5-HT could potentially recover flight. However, we discovered the opposite effect. 5-HT concentrations greater than 10 mM completely inhibited the mosquitoes’ ability to fly, therefore flight performance was not assessed at these concentrations. Concentrations of 10 mM 5-HT did not affect flight compared to the H_2_O alone treatment. The average distance for the 10 mM 5-HT treated group was 25.1 cm, while the 1.5 mM methiothepin-treated group was 41.38 cm. The group injected with the cocktail, however, landed at an average of 49.04 cm (Fig. 5c). While the impact of the cocktail was significantly different compared to the 10 mM 5-HT-treated group (p < 0.0001), this difference was not evident when compared to the 1.5 mM methiothepin-treated group (p = 0.4022). To further corroborate the 5-HT injection results, mosquitoes were treated with varying concentrations of fluoxetine, a selective-serotonin reuptake inhibitor. Similar trends were observed in flight behavior (Fig. 5d). Treatment with higher concentrations of the compound hindered the mosquitoes’ ability to fly. At 2.5 mM, flight was significantly undermined (distance average = 41.61 cm), while a higher concentration, 4 mM, completely abolished flight ability (distance average = 81.63 cm).

### Role of the serotonergic system in blood-feeding behavior

**Figure 6:**
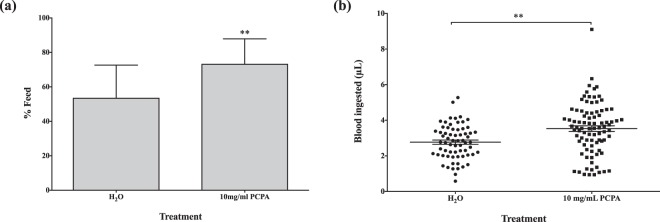
Effect of PCPA ethyl ester, an inhibitor of tryptophan hydroxylase, on blood-feeding. (**a**) Blood-feeding success of female *Ae. aegypti*. χ^2^: p = 0.0002. (**b**) Size of blood meal after treatment. Three independent experiments were conducted with a total n ~ 80 per treatment group. ^•^Represents the size of a blood meal for an individual mosquito. Data presented as mean ± standard error mean. Unpaired t-test: p = 0.0004.

The serotonergic system was manipulated to identify its role in blood-feeding behavior. To allow for the depletion of 5-HT, mosquitoes were fed a 10% sucrose solution containing varying concentrations (1, 5, 10, 25, and 60 mg/mL) of PCPA ethyl ester, a compound that inhibits the synthesis of tryptophan hydroxylase, a crucial enzyme in 5-HT synthesis. At the two highest concentrations (25 and 60 mg/mL), there was a 100% mortality rate after a three-day period. The groups provided with 1 and 5 mg/mL PCPA ethyl ester did not demonstrate any significant difference in feeding success or blood meal volume. In comparison to the control group, which ingested an average volume of 1.87μL of blood, the average blood meal size for 1 and 5 mg/mL PCPA-treated groups was 1.84 ± 0.16 μL (p = 0.89) and 2.33 ± 0.25 μL (p = 0.12), respectively. However, the blood feeding success was higher in the 10 mg/mL PCPA-treated group with 73.2% versus a 53.5% success rate in the control (p = 0.0002) (Fig. 6a). Furthermore, the volume of blood ingested by the treated group significantly exceeded that of the controls; the average blood meal size was 2.77 ± 0.12 μL for the H_2_O group, while the PCPA group imbibed an average of 3.53 ± 0.16 μL of blood (p = 0.0004) (Fig. 6b).

**Figure 7:**
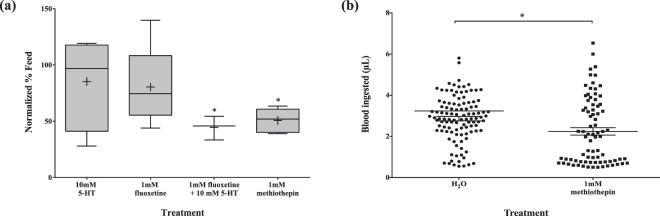
Manipulation of serotonergic system via chemical compounds and its effect on blood-feeding behavior in *Ae. aegypti*. (**a**) Blood-feeding success after treatment with 5-HT, fluoxetine (selective serotonin-reuptake inhibitor), combination of 5-HT and fluoxetine, and methiothepin (5-HT receptor antagonist). Data presented as box and whisker plots; the median value (line), mean value (+), interquartile range (box), and Tukey whiskers encompassing data within 1.5 fold of the interquartile range. Dunnett’s multiple comparisons test used to compare between treatment and control groups. *p-value < 0.02 (**b**) Size of blood meal after treatment with methiothepin. Three independent experiments were conducted with a total n ~ 80 per treatment group. ^•^Represents the volume of blood ingested by an individual mosquito. Data shown as mean ± standard error mean. Mann-Whitney test: p = 0.0015.

To test the oversaturation of the serotonergic system, exogenous 5-HT was introduced through treatment with 5-HT and fluoxetine, a selective 5-HT reuptake inhibitor. Mosquitoes microinjected with 10 mM 5-HT and 1 mM fluoxetine, independently, did not display a significant change in blood-feeding behavior. When presented with an artificial membrane feeder, approximately 80% of both treatment groups successfully blood-fed, when normalized to the untreated group. However, injection of 10 mM 5-HT and 1 mM fluoxetine, combined, effectively reduced blood-feeding behavior by approximately 55%. Paradoxically, microinjection of 1 mM methiothepin resulted in a similar phenotype, a diminished blood-feeding success rate. When normalized to the untreated group, an average of 50.7% methiothepin-treated mosquitoes blood-fed (p < 0.01) (Fig. 7a). Additionally, the mean blood volume consumption was significantly different between the two groups; the blood meal size was 2.86 μl versus 2.25 μl in the H_2_O and methiothepin-treated groups, respectively (p = 0.0015) (Fig. 7b).

## Discussion

Insect 5-HT GPCRs are localized in the central and peripheral nervous system and implicated in the regulation of processes such as feeding and locomotion via activation of downstream signaling pathways. Their role in such crucial behaviors for disease transmission warranted investigation into their capacity as targets for the insecticide pipeline. Calcium Gα_q_ assays were utilized to verify the inclusion of AGAP002232 and AGAP002229 in the 5-HT_2_ subfamily. Pharmacological characterization of AGAP002232 and AGAP002229 determined 5-HT to be the strongest agonist although 5-MT and tryptamine also demonstrated weak abilities for receptor activation. This appears to be a common finding in insects where not a single agonist has been identified to be as potent as the native ligand itself. In humans, however, 5-carboxamidotryptamine and 8-OH-DPAT display a strong affinity for 5-HT_7_ receptors, illustrating that differences in the pharmacological profiles exist and can be exploited for insecticidal purposes^22,23^.

The results of our study suggest that the serotonergic network plays a functional role in locomotion, but additional work is required to substantiate this finding. A reduction in flight performance was observed with the administration of fluoxetine, a selective serotonin-reuptake inhibitor (SSRI), high concentrations of 5-HT, and methiothepin, a non-selective 5-HT antagonist. Treatment with fluoxetine, and thus, an expected increase in serotonergic neurotransmission, has previously demonstrated to repress locomotion in both larval and adult *D. melanogaster*^24,25^. Similarly, microinjection of citalopram, another SSRI, into the thorax of *An. gambiae* resulted in severe flight defects and a prolonged time for flight recovery^26^. In *D. melanogaster*, the overexpression of tryptophan hydroxylase, a critical enzyme in the synthesis of 5-HT, significantly decreased locomotion^24^ while the generation of mutant enzymes led to increased levels of locomotion^25^. Paradoxically, we found that treatment with methiothepin also resulted in defects of flight performance. As an antagonist, this phenotypic outcome contradicts the expected reduction in 5-HT signaling.

Serotonergic neurotransmission also potentially modulates blood-feeding behavior. Evidence in multiple insect species including the honeybee, ant, locust, cockroach, and fly, demonstrates that 5-HT innervation causes a reduction in feeding^27,28^. Our fluoxetine and PCPA ethyl ester data favor this argument, though further work is required to validate it. We observed a reduction in feeding after administration of fluoxetine, while the opposing effect was detected with a diet supplemented with PCPA, a 5-HT depleting agent. A previous study examining the effects of the SSRI, paroxetine, on *Ae. aegypti* larval feeding behavior also described comparable findings^27^. While we expected methiothepin to induce a blood-feeding success similar to PCPA, we observed the contrary; microinjection of the drug curbed the mosquitoes’ success in attaining a blood meal. This finding complemented a previous study, which identified methiothepin as an anorectic agent from a small molecule drug screen in *D. melanogaster* larvae, and its antagonistic effect on the 5-HT_2a_ receptor^29^. However, these results do not align with our PCPA and fluoxetine data, and the true mechanism of action for methiothepin remains to be identified.

A plausible explanation for methiothepin’s opposing results displayed in locomotion and blood-feeding success is its potential antagonistic activity on the 5-HT_1_ autoreceptor in the pre-synaptic terminal. Although *in vitro* data demonstrates methiothepin to have a strong affinity for the 5-HT_2a_ receptor, this compound has also been shown to exert antagonistic activity on 5-HT_1a_, 5-HT_1b_, and 5-HT_7_ receptors in other invertebrates including *D. melanogaster*, *Apis mellifera, Tribolium castaneum*, and *Periplaneta americana*^23^. Typically, 5-HT_1_ autoreceptors initiate a negative feedback loop, prohibiting the further synthesis or release of the neurotransmitter. If methiothepin acts on this receptor subtype, an increase in 5-HT output would be expected, leading to higher serotonergic transmission, which is similar to fluoxetine’s mode-of-action^30^. Methiothepin’s non-selective nature, however, requires consideration because binding to both 5-HT_2_ and 5-HT_7_ receptors in the post-synaptic neuron should be expected as well. It is conceivable, though, that flooding the system with 5-HT could allow the native ligand to displace methiothepin on these receptors. Further understanding of the binding affinities and kinetics of 5-HT and methiothepin are required to shed light on this idea.

An alternative explanation for methiothepin’s paradoxical findings in our behavioral studies could be its antagonistic effects on the D_1_-like dopamine receptor, another biogenic amine system that has been implicated in regulating locomotion^19^. Studies in *A. mellifera* and *D. melanogaster* reveal that activation of dopaminergic neurons promote locomotion, while a reduction in dopamine inhibits this activity^31,32^. The dopaminergic and serotonergic system are tightly linked and evidence points towards the possibility of opposing action in the two, i.e. increasing 5-HT levels with decreasing dopamine. Finally, the excess of active 5-HT due to the fluoxetine’s inhibition of the serotonin reuptake transporter can be controlled by the dopaminergic system; the dopamine transporter can compensate by inactivating 5-HT, though with a low affinity^33^, and 5-HT can bind to dopamine receptors^26^. Under these circumstances, fluoxetine and methiothepin would act in a similar manner, by blockading the dopamine system from its natural ligand.

The results of this study suggest the serotonergic system’s involvement in flight performance and blood-feeding behavior, but there are limitations that require acknowledgement. While the prioritization of methiothepin materialized from *in vitro* work completed on *An. gambiae* 5-HT_2_ receptors, the *in vivo* behavioral experiments were performed on *Ae. aegypti*. This resulted from an assumption of similar folding and binding properties between 5-HT_2_ receptors in *Ae. aegypti* and *An. gambiae*. The 5-HT_2_ receptor homology between these two species is 38.3%, but ligand binding often occurs in the conserved transmembrane regions within GPCRs^34^. This led to the expectation that methiothepin would act in a similar manner in both *Ae. aegypti* and *An. gambiae*, and therefore, its use in the behavioral experiments. Additionally, although methiothepin, fluoxetine and PCPA have been shown to affect serotonin levels, we did not explicitly quantify serotonin levels in the individual mosquitoes after drug administration. Further experimentation is necessary to validate this assumption. Finally, although we are able to demonstrate that methiothepin acts on the *An. gambiae* 5-HT_2_ receptors *in vitro*, recognition in the complexity of the *in vivo* system is necessary. Methiothepin has the potential to act on a host of other targets, such as other aminergic signaling pathways, and isolating the primary target is difficult.

The complexity of the 5-HT system is evident in the vast network of enzymes, receptors, and transporters that tightly regulate endogenous levels. When homeostasis is lost, a multitude of mechanisms are triggered to regain control including, but not limited to, the modification of receptor expression or sensitivity, re-direction of the neural arborization network, and the binding to other biogenic amine transporters^24,26^. However, if mechanisms are unable to achieve control, downstream physiological functions can be impacted. Our qRT-PCR expression studies indicate that the various 5-HT GPCRs are present in all stages of the mosquito, but show elevated expression profiles in the adult stage versus the larval and pupal stages. In adult *Ae. aegypti*, our results suggest that dysfunction of the serotonergic system has the potential to adversely influence locomotion and blood-feeding behavior, which have considerable implications for global health. Reduced flight performance can negatively affect the mosquitoes’ ability to reach energy sources and execution of the “skip strategy” for oviposition. Moreover, blood-feeding not only directly influences disease transmission, but is critical for the egg development process^35,36^. Therefore, curtailing this behavior would not only reduce the burden of disease, but can also possibly abate the mosquito population. Considering these positive outcomes, the serotonergic system and more specifically, the 5-HT receptor, is a compelling target for the novel insecticide pipeline.

## Methods

### Bioinformatics

Due to the conserved amino acid sequence within the 5-HT GPCR family, BLAST-based sequence similarity and homology-based searches were conducted to identify the putative 5-HT receptor genes for *Ae. aegypti* and *An. gambiae*. The AaegL5 assembly of *Ae. aegypti* and AgamP4 assembly of *An. gambiae* hosted on VectorBase (www.vectorbase.org) were utilized. Further analysis was conducted with the GPCRHMM prediction tool^37^, which identified whether the amino acid sequence was a reasonable candidate for a GPCR based on topological characteristics, and provided a graphic of the protein’s structure. A rooted phylogenetic tree was also constructed with Geneious version 5.1.5 to provide a preliminary outlook on the relationships between 5-HT receptors and other biogenic amine receptors.

### Expression profiling

Total RNA was extracted from the immature larval and pupal stages, adult female and male whole bodies, and adult female head only with TRIzol Reagent (Invitrogen, Waltham, MA). Post DNase-treatment of RNA, cDNA was synthesized with SuperScriptIII reverse transcriptase (Invitrogen). Quantitative real-time PCR (qRT-PCR) was conducted with an ABI 7900 RT-PCR system, SYBRGreen (Applied Biosystems, Foster City, CA), 100 ng of cDNA, and final primer concentrations of 0.15 M. Housekeeping gene, 40 S ribosomal protein S7 (AAEL0009496, AGAP010592), was utilized as internal controls. Technical replicates were completed in triplicate and two independent experiments were conducted. Expression level of the genes was determined relative to the male whole body with the ΔΔCT method and expressed as 2^−ΔΔCt^. The following equations were used for the comparative quantification analysis. ΔC_t_ = C_t (gene of interest)_ − C_t (housekeeping gene)_. ΔΔC_t_ = ΔC_t (stage)_ − ΔC_t (whole male body)_.

### Cloning and expression of 5-HT receptors

Total RNA was isolated from a pool of 10 adult female mosquitoes with TRIzol Reagent (Invitrogen). The samples were treated with DNase I (Ambion Incorporated, Foster City, CA) to eliminate genomic contamination. The protein-coding region of the 5-HT receptors, flanked by short nucleotide sequences containing *SgfI* and *PmeI* restriction sites at the 5’ and 3’ ends, respectively, were amplified with PrimeStar polymerase (Takara, Japan), and the products were separated via agarose gel electrophoresis. The DNA products were extracted and purified with the QIAquick Gel Extraction kit (Qiagen, Hilden, Germany). After digestion with restriction enzymes *SgfI* and *PmeI*, the DNA products were cloned into the pCR 4-TOPO vector (Invitrogen). Colonies were isolated and prepared for Sanger sequencing by the University of Notre Dame Genomics and Bioinformatics Core Facility. Correct clones were then ligated to the mammalian pF9a CMV *hRluc-*neo Flexi® expression vector.

The GloResponse™ CRE-*luc2P* human embryonic kidney (HEK-293) reporter cells were incubated at 37 °C with an atmosphere of 5% CO_2_, and maintained in Dulbecco’s Modified Eagle Medium (DMEM) supplemented with 10% fetal bovine serum (FBS) and 50 mg/ml hygromycin B. Transfection of the HEK-293 cells with 1 μg of plasmid DNA was performed with Amaxa Nucleofector™ technology (Lonza, Basel, Switzerland) as per manufacturer’s instructions. A week post-transfection, selective pressure was applied with 400 mg/ml of G418 antibiotic to generate stable lines.

### Chemical compounds

Most of the chemical compounds were obtained from Sigma-Aldrich, Inc (St. Louis, MO) including serotonin hydrochloride (≥98%), selected biogenic amines (dopamine hydrochloride (≥98%), (±)-octopamine hydrochloride (≥95%), tyramine hydrochloride (≥98%), and histamine (97%)), agonists (5-methoxytryptamine (97%), α-methyl-5-HT, 1,(3-chlorophenyl)piperazine hydrochloride (99%), tryptamine hydrochloride (99%), quipazine maleate salt (≥98%)), antagonists (methiothepin mesylate salt (≥98%), yohimbine hydrochloride (≥98%), cyproheptadine hydrochloride sesquihydrate (99%), mianserin hydrochloride (≥98%), ketanserin tartrate salt (97%), and selective serotonin reuptake inhibitor, fluoxetine hydrochloride (≥98%). Stock solutions of the compounds were prepared with dimethyl sulfoxide (DMSO) for the *in vitro* experiments and ultrapure H_2_O (Invitrogen) for the *in vivo* experiments, and stored at −20 °C. 4-chloro-DL-chlorophenylalanine ethyl ester hydrochloride (98%) (PCPA) was purchased from Alfa Aesar (Ward Hill, MA) and prepared in 10% sucrose immediately before use.

### Luminescence assays

The reporter construct in the HEK-293 cells regulated the transcription of the *luciferase* gene in response to cAMP levels, while the R*luc*-Neo^r^ construct in the pF9a expression plasmid served as a normalizing agent. Cells suspended in 1% FBS/99% DMEM without phenol red were seeded in white 96-well plates at a density of 6.6 × 10^5^ per well, and immediately treated with varying concentrations of compounds. Following a 4-hour incubation at 37 °C with an atmosphere of 5% CO_2,_ the cells were prepared for a Dual-Glo luciferase assay according to the manufacturer’s instructions (Promega Corporation, Madison, WI). The luminescence units were measured on the SpectraMax L 96 microplate luminometer (Molecular Devices, Sunnyvale, CA) and normalized to the cell number.

### Calcium assays

1 × 10^6^ cells per well were seeded into a 96-well black all-clear bottom plate and incubated overnight in 10% FBS/90% DMEM at 37 °C with an atmosphere of 5% CO_2._ The following day, 2X Fluo-4 Direct calcium dye (Molecular Devices LLC, San Jose, CA) was added to each well and incubated in the same conditions for 45 minutes before the assay was completed. At this time, serial dilutions of the test compounds were prepared in a 96-well clear flat bottom plate. Fluorescence levels were determined with a FlexStation3 microplate reader, with excitation and emission wavelengths set to 485 and 525 nm, respectively (Molecular Devices LLC). Basal fluorescence levels were measured for 17 seconds, at which time, 25 μl of the agonist compound was added. Fluorescence measurements continued to be recorded for a total of 120 seconds at 2-second intervals. When testing antagonists, cells were treated with the compound prior to determination of fluorescence levels.

### Mosquito rearing

*Ae. aegypti* (strain: Liverpool) mosquitoes were reared in an insectary maintained at constant environmental conditions: 26 °C, 85% relative humidity, and a 16-hour light and 8-hour dark photoperiod cycle with one-hour dusk and dawn periods. Larvae were raised in pans and provided with liver powder for nutrition. Adults were maintained in plastic cages and fed *ad libitum* with a 10% sucrose solution. Adult female mosquitoes were blood-fed on a rat for colony maintenance.

### Mosquito treatment

#### Microinjection

Stock solutions of the compounds were diluted to the appropriate concentrations in 1X phosphate buffer solution (PBS). To account for potential solvent effects, an ultrapure H2O control group was included in every independent experiment. Female mosquitoes were either cold- or CO_2_-anesthetized. A Nanoject II Auto-Nanoliter Injector (Drummond Scientific Company, Broomall, PA) was then utilized to microinject 69 nL of compound into the spiracle.

#### Feeding

Sucrose solution was withheld from newly-emerged mosquitoes for two days. On the third day, the mosquitoes were presented with a cotton ball saturated with 400 μL of a 10% sucrose solution laced with compound or the respective control for five days. The cotton ball was exchanged every other day with a fresh one.

### Flight performance

A flight cylinder was assembled as described previously^21^. The polyacrylamide sheet was coated with Tangle-Trap Sticky Coating (The Scotts Company LLC, Marysville, OH) and allowed to sit overnight before insertion into the flight cylinder. Adult mosquitoes were anesthetized on a CO_2_ pad and microinjected with the compound or ultrapure H_2_O control. Post-injection, they were permitted to recover in a fly vial for 30 minutes before being ejected into the flight cylinder. After each trial, the distance between the top of the cylinder and the trapped mosquito was recorded. Mosquitoes captured in the fly vial at the bottom of the cylinder were considered to be incapable of flying and a distance of 91.5 cm was recorded.

### Blood-feeding assays

Seven to ten-day old female mosquitoes were treated with compound or appropriate control through microinjection post cold-anesthetization or ingestion. Two mL of human O^+^ blood (BioChemed Services, Winchester, VA) was utilized to cover the concave surface of an inverted baby food jar. Hog intestine sealed the opening to simulate the puncturing of a host’s skin. The feeding membrane was placed on the top of a mosquito cage, and hot water was poured into the jar to warm the blood. After a 45-minute feed, the mosquitoes were immediately preserved at −20 °C. The blood meal size was determined for each mosquito via hemoglobinometry as previously described^38^.

### Statistical analyses

Luminescence and calcium assay data were normalized and fitted with a non-linear least squares regression to generate dose-response curves, EC_50_ and IC_50_ values. Unpaired t-tests or Dunnett’s multiple comparisons test were performed accordingly to compare EC_50_ or IC_50_ values. Locomotion was measured based on the distance required for flight recovery. Data were compared with the Kruskal-Wallis and Dunnett’s multiple comparison tests after the Kolmogorov-Smirnov normality test was conducted. In the blood-feeding experiments, the percentage of blood-fed mosquitoes was calculated for each treatment group. For each biological replicate, the treatment groups were normalized with respect to the untreated group. Dunnett’s multiple comparison tests were utilized to determine statistical difference between normalized data. Size of bloodmeals were compared with an unpaired t-test or Mann-Whitney test after normality was evaluated with the Kolmogorov-Smirnov test. All analyses were performed utilizing GraphPad Prism version 7.00 for Mac OS X, GraphPad Software, La Jolla California USA, www.graphpad.com.

## Supplementary information

Supplemental Information

Supplemental Information

Supplemental Information

Supplemental Information
